# Argan oil prevents prothrombotic complications by lowering lipid levels and platelet aggregation, enhancing oxidative status in dyslipidemic patients from the area of Rabat (Morocco)

**DOI:** 10.1186/1476-511X-12-107

**Published:** 2013-07-20

**Authors:** Adil Haimeur, Hafida Messaouri, Lionel Ulmann, Virginie Mimouni, Azelarab Masrar, Abdelmjid Chraibi, Gérard Tremblin, Nadia Meskini

**Affiliations:** 1 IUT Département Génie Biologique, Université du Maine, PRES L’UNAM, EA 2160 MMS (Mer, Molécules, Santé), Faculté des Sciences et Techniques, Le Mans, Laval, France; 2 Université Hassan II, Equipe Nutrition, Environnement, Santé. Laboratoire de Virologie, Microbilogie, Qualité/Ecotoxicologie et Biodiversité, Faculté des Sciences et Techniques, Mohammedia, Morocco; 3 Centre Hospitalier Universitaire Ibn Sina, Service d’Endocrinologie Diabétologie et Nutrition, Rabat, Morocco; 4 Centre Hospitalier Universitaire Ibn Sina, Laboratoire d’Hémostase, Faculté de Médecine et de Pharmacie, Rabat, Morocco

**Keywords:** Platelet aggregation, Argan oil, Dyslipidemia, Oxidative stress

## Abstract

**Background:**

It is now established that patients with hyperlipidemia have a high risk of atherosclerosis and thrombotic complications, which are two important events responsible for the onset and progression of cardiovascular disease. In the context of managing dyslipidemia by means of dietary advice based on the consumption of argan oil, we wanted to investigate the effect of virgin argan oil on plasma lipids, and for the first time, on the platelet hyperactivation and oxidative status associated with dyslipidemia. This study concerns patients recruited in the area of Rabat in Morocco.

**Methods:**

39 dyslipidemic (79% women) patients were recruited for our study in the area of Rabat in Morocco. They were randomly assigned to the two following groups: the argan group, in which the subjects consumed 25 mL/day of argan oil at breakfast for 3 weeks, and the control group in which argan oil was replaced by butter.

**Results:**

After a 3-week consumption period, blood total cholesterol was significantly lower in the argan oil group, as was LDL cholesterol (23.8% and 25.6% lower, respectively). However, the HDL cholesterol level had increased by 26% at the end of the intervention period compared to baseline. Interestingly, in the argan oil group thrombin-induced platelet aggregation was lower, and oxidative status was enhanced as a result of lower platelet MDA and higher GPx activity, respectively.

**Conclusions:**

In conclusion, our results, even if it is not representative of the Moroccan population, show that argan oil can prevent the prothrombotic complications associated with dyslipidemia, which are a major risk factor for cardiovascular disease.

## Background

Cardiovascular disease (CVD) is the main cause of increasing mortality worldwide. In 2008, heart disease accounted for 23% of deaths in Morocco [1]. Dyslipidemia, the main risk factor for CVD, is a heterogeneous metabolic disorder involving multiple etiologies; it is commonly characterized by increased triglycerides, low-density lipoprotein (LDL) cholesterol and apolipoprotein B (apo-B) levels, and reduced plasma high-density lipoprotein (HDL) cholesterol [2]. These components of dyslipidemia confer an increased risk for atherosclerosis and thrombosis, which are two important events responsible for the onset and progression of CVD [3]. It is now established that patients with hyperlipidemia have a high risk of atherosclerosis and thrombotic complications [4-6]. Several mechanisms have been suggested by which alterations in plasma lipid levels may promote atherosclerosis [7-9]. One major determinant of the prothrombotic state associated with hypercholesterolemia appears to be related to enhanced platelet activation. Indeed, platelets are known to play a fundamental role in atherogenesis and in the pathology of atherothrombotic disorders [10]. *In-vivo* platelet activation has been reported in type-IIa hypercholesterolemic patients [11], suggesting that high levels of oxidized LDL, via changes in the composition of platelet membrane phospholipids and cholesterol, may increase the platelet reactivity associated with enhanced thromboxane A_2_ (TxA_2_) biosynthesis. The involvement of blood platelet activation in the onset of atherosclerosis is well established. It has long been recognized that platelets are involved in the late thrombotic complications of atherosclerosis plaque due to erosion of the endothelium or rupture of the fibrous cap that covers the lipid core within the plaque [12]. The association of platelet activation with acute CVD justifies anti-platelet therapy as a standard treatment for those at high risk of atherothrombosis.

Several epidemiological, clinical, and experimental studies have established that diet can have beneficial effects on the cardiovascular system, and can therefore be considered to provide an effective therapeutic tool for preventing several CVD risk factors, such as dyslipidemia, platelet hyper-reactivity, and diabetes [13,14]. As far as ingested lipids are concerned, the influence of their quality and quantity has often been demonstrated in human and animal models [15]. Haimeur et al. [16] showed that a high fat diet consumption increased the incidence of CVD in rats, by increasing blood lipids level, platelet aggregation and hepatic oxidative stress, however these risk factors of CVD were lower in rats fed with a high fat diet supplemented with a freeze-dried of *Odontella aurita*, a marine diatom rich in omega-3 (eicosapentaenoic acid) and antioxidants molecules.

Argan oil is a natural vegetable oil that has been widely used in both the daily diet and folk medicine in Morocco. It is extracted from the kernels of *Argania spinosa*, an endemic tree growing in the south-western region of Morocco. The main traditional use of this edible oil is for nutritional purposes by consuming it directly on toast, generally during breakfast [17]. This oil is rich in unsaturated fatty acids (80%) principally oleic and linoleic acids (44.8% and 33.7%, respectively) [18]. Interestingly, the unsaponifiable fraction of argan oil (1% of the oil constituents) contains mainly antioxidant compounds, such as tocopherols, especially γ-tocopherol, which are present in higher portions than in olive oil (637 mg/kg versus 258 mg/kg of total tocopherols, respectively) (Table 1) [18]. As could be expected from its interesting composition, argan oil has demonstrated its pharmacological effects in several studies. Initially demonstrated in rats [19-22], the hypocholesterolemic potency of argan oil in humans has also been demonstrated by a cohort study of 60 healthy men [23]. Numerous nutritional studies have also shown that argan oil lowers LDL cholesterol and has antioxidant properties; these properties have been demonstrated in a cohort study involving 96 people [24]. Here, the subjects who consumed argan oil on a regular basis presented with significantly lower levels of plasma LDL cholesterol and lipoprotein A Lp (a), and lower levels of plasma lipoperoxides (thiobarbituric acid reactive substances, TBARS) [24]. Argan oil also increases HDL cholesterol and lowers triglyceride levels in men [25], and therefore regular argan oil consumption could potentially prevent diabetes [26]. Furthermore, when rats were fed for 4 weeks with 10 mL/kg/day of argan oil, the thrombin-induced aggregation of isolated platelets was significantly inhibited (36%), however, the tail bleeding time remained unchanged [27]. Recently, Mekhfi et al. [28] have reported that argan oil has antithrombotic activity in rats, which could be related to its antiplatelet rather than its anticoagulant activity.

**Table 1:** **Chemical composition of argan oil**[18]

**Fatty acids**	**(%)**
C 16:0	13.4
C 18:0	5.1
C 18:1	44.8
C 18:2	35.7
C 18:3	0.1
**Sterols mg/100 g oil**	
Schottenol	142
Spinasterol	115
Stigmasta-8,22-dien-3-ol	9
Others	29
**Tocopherols mg/Kg oil**	
α-tocopherol	35
δ-tocopherol	122
γ-tocopherol	480
**Phenols μg/Kg oil**	
Vanillic acid	67
Syringic acid	37
Ferulic acid	3147
Tyrosol	12

Argan oil has already been reported to be a hypolipidemic factor in both human and animal models without producing any adverse effects [20,24]. However, the anti-aggregant effect of argan oil has so far only been described *in-vitro* in rats [27]. Thus the originality of the study reported here, is investigating the protective effect of argan oil against platelet disorders, such as hyper-activity and oxidative stress in dyslipidemic patients. According to the low sampling of patient recruited in the area of the city of Rabat, our exploratory study is not significant of the moroccan population.

## Methods

### Patients

This study was conducted on dyslipidemic patients attending the Endocrinology Department of the Ibn Sina University Hospital, Rabat, Morocco. The inclusion criteria was patients with hypercholesterolemia or/and hypertriglyceridemia. The exclusion criteria included myocardial infarction, the presence of hepatic or renal disease, the use of anti-aggregant drugs (aspirin, thienopyridines) or of lipid-lowering drugs (statins), unusual diet (low or high fat or carbohydrate diets), or taking hormone therapy during the previous 6 months. The protocol and objectives of this study were explained to the participants in detail. Before the recruitment, patients give their consent orally. The study protocol was approved by the Institutional Ethics Committee of the Endocrinology, Diabetology, and Nutrition Department, Ibn Sina University Hospital, Rabat, Morocco. Among the 78 patients who consulted, a total of 39 dyslipidemic patients were selected on the basis of the inclusion criteria. All the patients included underwent a thorough physical examination in the Service at 3 consultations during the study.

### Study design

This study was conducted according to the protocol described by Derouiche et al. [25]. The study design consisted of two diet periods. The first diet period (stabilization period) lasted 2 weeks during which all the patients consumed 20 g/day of butter on toasted bread for breakfast, and both argan oil and olive oil were eliminated from their diet. This stabilization period was intended to ensure that all the patients had a similar fat intake and stable dyslipidemia parameters before starting the study. In the second diet period (nutritional intervention), which lasted 3 weeks, the patients were randomly assigned to one of two groups: the first group (18 patients) received the 20 g/day butter diet (control group: C); the second group (21 patients) received 25 mL/day of virgin argan oil instead of the butter with the toasted bread for breakfast (argan oil group: AO). The daily eating habits of all the patients were investigated by means of a comprehensive food questionnaire covering all foods commonly consumed in Morocco. During the nutritional intervention period, all patients followed a lifestyle and diet recommended by the guidelines for the management of dyslipidemia [29]. The argan oil supplied to the patients was all purchased from the same source, and was extracted by an industrial process [30].

### Blood collection

During the study, the patients provided 3 blood samples. The first was collected intravenously in vacuum tubes after they had fasted for 12 h, and was used to confirm their dyslipidemia by serum lipid analysis. At the end of the stabilization period, the second sample was collected intravenously into two tubes: the first was a vacuum tube containing EDTA, and this blood was used for serum lipid analysis, and the other was a tube containing citric phosphate dextrose (CPD) anticoagulant (citric acid: 15.6 mM; sodium citrate: 89.4 mM, monosodium phosphate: 16.1 mM and dextrose: 128.7 mM) in a ratio of 1/7 v/v CPD/blood, and this was used for platelet analysis (platelet aggregation test, basal TxB_2_ determination and platelet oxidative status). The third blood sample was similar to the second and was collected at the end of the nutritional intervention.

### Anthropometric and serum lipid parameters

Demographic and anthropometric parameters (age, sex, weight, body mass index (BMI), blood pressure) were recorded for the patients at baseline. Serum total cholesterol (TC) and HDL-cholesterol levels were determined using commercial enzymatic kits (Randox cholesterol enzymatic kit and Randox HDL cholesterol precipitant for cholesterol and HDL-cholesterol, respectively, Crumlin, Co. Antrim, UK) according to Richmond’s colorimetric procedure [31]. Serum triglyceride levels were determined using a commercial kit (Randox, Triglyceride Enzymatic Kit, Crumlin, Co. Antrim, UK) according to Trinder’s colorimetric method [32]. The LDL-cholesterol level was calculated by Friedewald’s formula [33]. LDL cholesterol (g/L) = total cholesterol (g/L) – HDL cholesterol (g/L) – triglycerides/5 (g/L).

### Platelet aggregation

Platelets were isolated as previously described by Lagarde et al. [34]. Briefly, blood was centrifuged at 200 x g for 15 min at 20°C to obtain platelet-rich plasma (PRP). PRP was then acidified to pH 6.4 with 0.15 M citric acid, and immediately centrifuged at 900 x g for 10 min at 20°C. Platelet pellets were resuspended in Tyrode/HEPES buffer solution (137 mM NaCl, 2.7 mM KCl, 11.9 mM NaHCO_3_, 0.41 mM NaH_2_PO_4_, 1 mM MgCl_2_, 5.5 mM glucose, 5.0 mM HEPES, pH 7.35). Platelet concentration was adjusted to 300 000 /μL before aggregation assay. The isolated platelets were then used to determine platelet aggregation by turbidimetry [35] using a PACKS 4 aggregometer (HELENA, TX, USA). Aggregation tests were performed at 37°C in cuvettes stirred at 1000 rpm. Isolated platelets were stimulated by exposure to thrombin (Sigma-Aldrich, Saint-Quentin Fallavier, France) 0.2 U/mL. The light transmission was recorded for 3 min after platelet stimulation. The platelet aggregation was quantified from the maximum change in light transmission through a washed platelet suspension, expressed as a percentage of the light transmission through the blank (Tyrode buffer).

### Platelet MDA measurement

Platelet lipid peroxidation was evaluated by measuring the malondialdehyde (MDA) level according to the method described by Ohkawa [36]. To 500 μL of platelet solution were added 100 μL of 8.1% sodium dodecyl sulfate (SDS), 750 μL of acetic acid 20% at pH 3.5, 750 μL of thiobarbituric acid (TBA) 0.8%, and distilled water to make up the volume to 2.5 mL. The tubes were maintained in a water bath at 95°C for 60 min. The tubes were then immediately cooled in ice. 500 μL of distilled water and 2.5 mL of a solution of *n*-butanol and pyridine (15:1 v/v) were then added to each tube. The tubes were shaken vigorously before being centrifuged at 1000 x g for 10 min. The organic phase, corresponding to the upper layer, was aspirated and its color intensity measured at 532 nm. A standard range was prepared using a 1,3,3,3- tetramethoxypropane (Sigma-Aldrich, Saint-Quentin Fallavier, France) solution.

### Platelet glutathione peroxidase activity

The glutathione peroxidase (GPx) activity was determined in isolated platelets according to the Paglia and Valentine method [37], as modified by Chaudiére and Gérard [38]. Briefly, 100 μL of platelet lysate was added to a final volume of 1.5 mL of a solution containing Tris-HCl/EDTA (50 mM/0.1 mM) pH 7.6, NADPH, H^+^ (0.14 mM), reduced glutathione (GSH) (2 mM) and 0.7 U/mL of GSSG-reductase. The mixture was incubated at 37°C for 3 min. The reaction was started by adding 50 μL tertiary butyl hydroperoxide (*t*-BH) (0.2 mM). The change in absorbance was recorded at 340 nm for 5 min at 5 s intervals. An appropriate control without the platelet sample was run simultaneously. GPx activity was expressed in nmoles of hydroperoxide reduced per min and per mg protein.

### Thromboxane B_2_ measurement

To determine the platelet thromboxane B_2_ (TxB_2_) level, isolated platelets were subjected to three successive freeze/thaw cycles to release the cell contents. The baseline TxB_2_ level was measured by enzyme immunoassay (EIA) Kit (Enzo-Life Sciences, Exeter, U.K) according to the Manufacturer’s instructions.

### Assay of proteins

Platelet total proteins were determined by the Bradford colorimetric method [39] using Biorad reagent and bovine serum albumin as standard (Sigma-Aldrich, Saint-Quentin Fallavier, France).

### Statistical analysis

All values were expressed as the mean ± SD. After analysis of variance, the mean values were compared using Fisher's least significant difference test (Statgraphics Plus 5.1, Manugistics Inc., Rockville, MD, USA). P < 0.05 was accepted for significant differences.

## Results

### Baseline anthropometric parameters

The baseline characteristics of the patients, including their sex and age as well as the risk factors associated with atherosclerosis diseases, are presented in Table 2. At the beginning of the intervention period, the data showed that both groups displayed abnormal blood lipid markers characterized by hypercholesterolemia and hypertriglyceridemia. According to the International Diabetes Federation (IDF) definition [40], all of the patients exhibited at least two components of metabolic syndrome (MS). In addition to hypertriglyceridemia and hypercholesterolemia, the most frequent component of the MS in our patients was central obesity.

**Table 2:** Baseline anthropometrical and biological characteristics of the study groups

**Characteristic**	**Control group**	**Argan oil group**
**Sex (W/M)**	14/4	17/4
**Age (years)**	51 ± 9	55 ± 8
**Weight (Kg)**	74.9 ± 13.0	72.3 ± 9.4
**SBP (mmHg)**	11.9 ± 1.3	12.4 ± 0.9
**DBP (mmHg)**	6.7 ± 0.9	6.9 ± 0.7
**BMI (Kg/m2)**	29.6 ± 5.0	29.7 ± 4.9
**TC (g/L)**	2.53 ± 0.3	2.41 ± 0.4
**TG (g/L)**	1.62 ± 0.1	1.73 ± 0.2
**HDL-chl (g/L)**	0.45 ± 0.07	0.50 ± 0.05
**LDL-chl (g/L)**	1.13 ± 0.2	1.35 ± 0.3

### Serum lipid parameters

**Figure 1:**
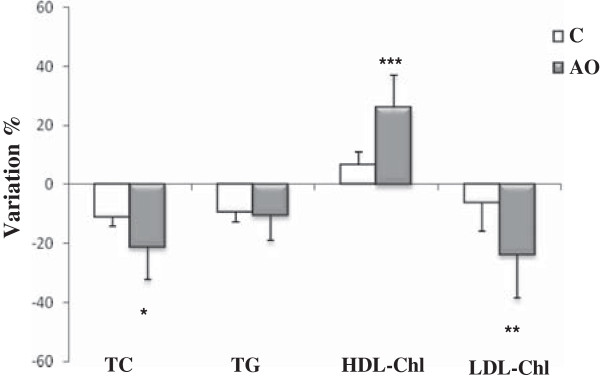
**Variations in different plasma lipid markers after 3 weeks of nutritional intervention with argan oil.** Data are the percentage variation of the lipid parameters between the two periods. Comparisons are made between the variation in the two groups of patients (C: control group; AO: argan oil group). TC: total cholesterol; TG: triacylglycerol ; HDL-Chl : high density lipoprotein cholesterol; LDL-Chl: low density lipoprotein cholesterol. After a one-way ANOVA, Student-Newman-Keuls multiple test, a comparison was made between the two groups of patients. ***: *p* = 0.01; **: *p* = 0.02; *: *p* = 0.04.

After the three weeks of the nutritional intervention period, a significant improvement in atherogenic lipid parameters was found in the AO group patients as compared to the values found before the nutritional intervention period (Figure 1). The data showed significantly (*p* = 0.04) lower total cholesterol and LDL-cholesterol levels (*p* = 0.02) and significantly (*p* = 0.01) higher HDL-cholesterol levels in the group consuming argan oil than in the control group of patients (23.8% versus 13.5%, 25.6% versus 10.4%, and 26% versus 6.6%, respectively). However, there was no difference in triglyceride levels between the two groups after the three weeks of the intervention period.

### Platelet aggregation and TxB_2_ production

**Figure 2:**
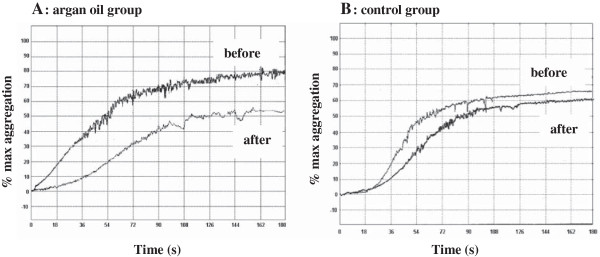
**Original traces showing effect of argan oil on platelet aggregation.** Isolated platelets were stimulated by exposure to 0.2 U/mL thrombin. before: before treatment; after: after treatment.

**Figure 3:**
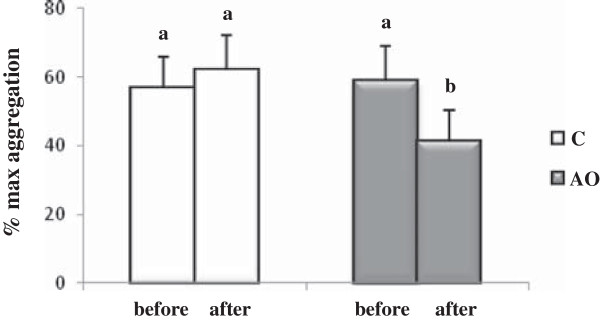
**Platelet aggregation after 3 weeks of nutritional intervention with argan oil.** Washed platelets were stimulated with thrombin (0.2 U/ml). Comparisons are made between the % platelet aggregation before and after the intervention period in the two groups. C: control group; AO: argan oil group. After a one-way ANOVA, Student-Newman-Keuls multiple comparison test, results are arranged in increasing order from left to right a > b (*p* < 0.05).

Platelet hyperactivity is associated with an increase risk of adverse cardiovascular outcomes. We evaluated the platelet function of patients by studying the platelet response to aggregation agonist stimulation. The thrombin-induced aggregation exemple is shown in the Figure 2. The results obtained after the three weeks of the nutritional intervention period showed that the percentage thrombin-induced platelet aggregation was significantly (*p* = 0.03) lower in the AO group than in the control group (44.5% versus 62.6%) at the end of the intervention period (Figure 3).

**Figure 4:**
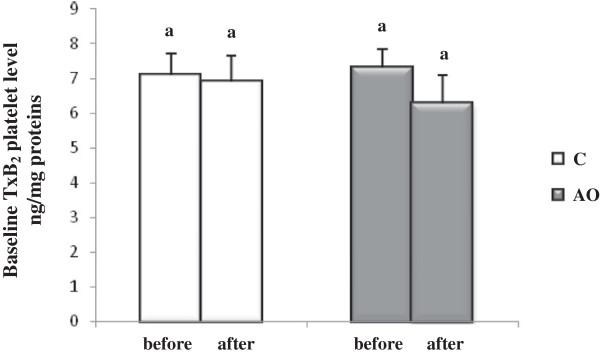
**Platelet thromboxane B**_**2 **_**(TxB**_**2**_**) level after 3 weeks of nutritional intervention with argan oil.** Comparisons are made between the platelet TxB_2_ level before and after the intervention period in the two groups. C: control group; AO: argan oil group. After a one-way ANOVA, Student-Newman-Keuls multiple comparison test, results are arranged in increasing order from left to right a > b (*p* < 0.05).

On another hand, the baseline concentration of TxB_2_, the stable catabolite of TxA_2,_ tended to be lower in the AO group than in the control group after the three weeks of the intervention period, but this difference was not statistically significant (*p* = 0.1) (Figure 4).

### Platelet oxidative status

**Figure 5:**
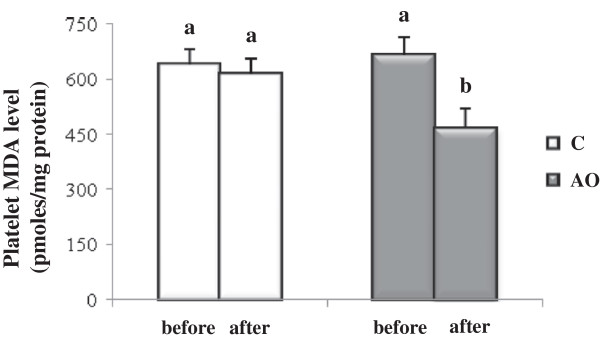
**Platelet malondialdehyde (MDA) level after 3 weeks of nutritional intervention with argan oil.** Comparisons are made between the platelet MDA level before and after the intervention period in the two groups. C: control group; AO: argan oil group. After a one-way ANOVA, Student-Newman-Keuls multiple comparison test, results are arranged in increasing order from left to right a > b (p < 0.05).

**Figure 6:**
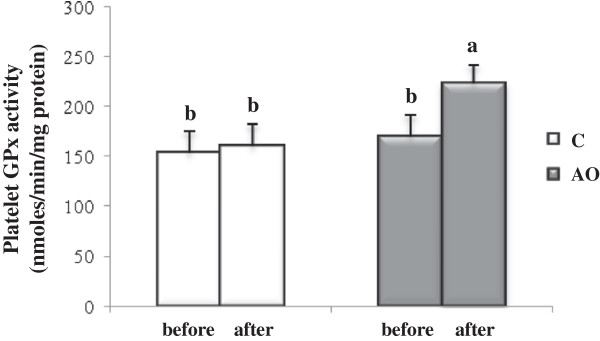
**Platelet glutathione peroxidase (GPx) activity after 3 weeks of nutritional intervention with argan oil.** Comparisons are made between the platelet GPx activity before and after the intervention period in the two groups. C: control group; AO: argan oil group. After a one-way ANOVA, Student-Newman-Keuls multiple comparison test, results are arranged in increasing order from left to right a > b (p < 0.05).

The results for the platelet oxidative status are shown in Figures 5 and 6. After the three weeks of the intervention period, the data showed that platelet lipid peroxidation levels were lower in patients consuming argan oil, with an MDA level about 34% lower (*p* = 0.02) than in the control group at the end of the intervention period (Figure 5). This result was correlated with the activity of platelet GPx, the main enzyme catalyzing the reduction of lipid hydroperoxides to the corresponding alcohols (Figure 6). Interestingly, at the end of the intervention period, the platelet GPx activity in the AO group increased by 22% (*p* = 0.02) when compared to the control group.

## Discussion

Hypercholesterolemia and platelet hyperactivity are associated with an increased risk of adverse cardiovascular outcomes (coronary artery disease, hypertension). Argan oil is rich in unsaturated fatty acids, and also contains a non-glyceride fraction characterized by the presence of sterols and antioxidant components, which gives it interesting biological and pharmacological properties. The present investigation was undertaken to assess the potential platelet anti-aggregant and antioxidant effects of argan oil on dyslipidemic patients. This clinical study involved evaluating the effects of argan oil after a three-week period during which consuming argan oil on toasted bread for breakfast was compared with consuming butter on toasted bread.

Interestingly, our results showed that argan oil appeared to be effective in preventing hypercholesterolemia in dyslipidemic patients. They show that after three weeks of argan oil consumption, circulating total cholesterol and LDL-cholesterol lowered by about 24% and 25%, respectively, compared to the values obtained before the nutrition intervention period. In addition, patients who consumed argan oil had a higher plasma HDL-cholesterol levels than control patients. However, argan oil had no effect on circulating triglyceride levels after three weeks of intervention, whereas Derouiche et al. [25] reported that plasma triglyceride levels decreased significantly by 17.5% in healthy people consuming about 28 mL/day of argan oil over a 3-week period. Indeed, a lipid-lowering effect of argan oil has been reported in several other studies involving either animals or healthy male subjects [41-43], but our results demonstrate for the first time the hypocholesterolemic effect of argan oil on dyslipidemic patients. There is now abundant evidence that diets high in unsaturated fatty acids have a hypocholesterolemic effect when substituted for diets high in saturated fatty acids [44,45]. This has been showed by the beneficial effects of the “Mediterranean diet”, which is attributed, besides the effects of fibers, complex carbohydrates, vegetable proteins etc, to its high content of olive oil, which is an important source of monounsaturated fatty acids (MUFAs) (73% of oleic acid), whereas sunflower oil has a high content of polyunsaturated fatty acids (PUFAs) (61.6% of linoleic acid) [46]. However, argan oil is also characterized by containing balanced proportions of MUFAs and PUFAs (44.8% oleic acid and 33.7% linoleic acid) [18]. Linoleic acid (C18: 2n − 6) is an essential fatty acid, and is a precursor in the biosynthesis of arachidonic acid (C20: 4n − 6). Arachidonic-derived fatty acids, particularly γ-linolenic acid, are known to reduce the total cholesterol, VLDL, and LDL-cholesterol levels in human and rat serum [47,48]. In addition to unsaturated fatty acids, other minor components of argan oil, such as plant sterols, may also be implicated in the hypocholesterolemic effect of this oil. Phytosterols differ from cholesterol by having ethyl or methyl groups at carbon-24 and/or a double bond at carbon-22. These features increase their hydrophobicity and reduce their absorption [49]. Phytosterols compete with endogenous cholesterol for space within bile salt micelles in the intestinal lumen, thereby reducing cholesterol absorption [50,51]. A recent, randomized, placebo-controlled, crossover trial reported that incorporating softgel capsules containing esterified plant sterols/stanols 1.8 g/day into the National Cholesterol Education Program (NCEP) Therapeutic Lifestyle Changes diet, produced favorable changes in atherogenic lipoprotein and cholesterol levels in subjects with hypercholesterolemia [52]. These results corroborate those found in several studies conducted with argan oil [25,41-43]. Furthermore, the cholesterol-lowering effect of argan oil observed in our study could be related to a synergistic effect of unsaturated fatty acids and sterols, the main constituents of this oil.

Accumulating evidence indicates that oxidative stress plays a significant role in the onset of atherosclerosis [53], and that platelet activation is an important contributing factor in the process of atherosclerosis and its thrombotic complications. In this study, we investigated the effect of argan oil on platelet aggregation, TxB_2_ release, and platelet oxidative status in dyslipidemic patients. Our results showed that argan oil produced an anti-aggregant effect after it had been consumed for three weeks instead of butter at breakfast. This anti-aggregant effect was demonstrated by a 29% decrease in thrombin-induced aggregation and a tendency towards lower baseline TxB_2_ levels in the AO group than in controls. *In-vitro*, an inhibition of thrombin- or epinephrine-induced aggregation by up to 50% was obtained in isolated rats platelets with a dose of 0.5% of argan oil [27]. At least two mechanisms are probably involved by which the fatty acids regulate platelet function: modulation of the pool of precursor fatty acids and their competitors, with subsequent effects on eicosanoid metabolism, and changes in cell membrane fluidity, resulting in changes in the activity and/or efficiency of platelet agonist receptors [54,55]. The chemical composition of argan oil is dominated by unsaturated fatty acids (80%), principally oleic and linoleic acids. These unsaturated fatty acids are known to be precursors of pro-thrombotic and pro-inflammatory eicosanoids; so the anti-aggregant effect observed could be related to the unsaponifiable fraction of argan oil (1% of the oil constituents), which is richer in tocopherols than olive oil. It also contains other important compounds, such as phenols, principally ferulic and syringic acids, which have already been reported to be anti-aggregants [56]. However, several studies have related the anti-aggregant effect of linoleic acid to the synthesis of prostaglandins E1 (PGE_1_) and prostacyclins I_2_ (PGI_2_), which share the same receptor in platelet membranes and inhibit platelet aggregation by increasing cyclic AMP (cAMP) through the activation of receptor-linked adenylate cyclase [57,58]. Furthermore, most oxidant-induced alterations are influenced by the nature of the cellular redox status. It is known that the increased lipid peroxidation observed in some redox-sensitive pathological states (such as dyslipidemia and diabetes) [59] is closely associated with impairment of the antioxidant defenses against peroxides, i.e. decreased activity of GPx [60], the main enzyme that catalyzes the reduction of lipid hydroperoxides to the corresponding alcohols. The results obtained on the platelet oxidative status showed an antioxidant effect of argan oil after three weeks of intervention. This antioxidant effect was manifested by lower platelet MDA levels and higher platelet GPx activity in patients who had consumed argan oil for three weeks compared to control patients who had not. In spite of the high antioxidant content of argan oil, little is known about its mechanism of action. *In-vitro* investigations of the antioxidant effect of argan oil have reported that incubating LDL with tocopherols, sterols, and phenolic extracts of argan oil significantly prolonged the lag-phase of LDL peroxidation [61]. Also, phenolic extracts have been shown to lower the rate of lipid peroxidation, and reduce the disappearance of vitamin E in a concentration-dependent manner [61]. Other *ex-vivo* studies have investigated whether the consumption of argan oil could improve antioxidant status in healthy male subjects [23]. Indeed, polyphenols, tocopherols, and sterols could all act as powerful antioxidants via several mechanisms: scavenging of peroxy radicals that break the peroxidation chain reaction; chelating free Cu^2+^ to form redox-inactive complexes, thus reducing metal-catalyzed oxidation; and inhibiting the binding of Cu^2+^ to apolipoproteins and subsequently preventing the modification of the amino acid apo-B protein residue [62].

In conclusion, our study shows that argan oil intake for a period of three weeks reduces the risk factors for prothrombotic complications by decreasing plasma cholesterol levels and preventing the platelet hyperactivity and oxidative stress associated with dyslipidemia. Despite the limitations of our study due to the difficulty of collecting sufficient blood to perform all the platelet analysis, these new data are of considerable relevance to the management of dyslipidemia, which is a major risk factor for CVD. Finally, argan oil could be part of the dietary advice provided in the context of the nutritional management of atherosclerotic CVD.

## Abbreviations

CVD: Cardiovascular disease; LDL: Low-density lipoprotein; HDL: High-density lipoprotein; apo-B: Apolipoprotein B; TxA2: Thromboxane A_2_; TBARS: Thiobarbituric acid reactive substances; CPD: Citric phosphate dextrose; BMI: Body mass index; PRP: Platelet-rich plasma; MDA: Malondialdehyde; GPx: Glutathione peroxidase; IDF: International Diabetes Federation; MS: Metabolic syndrome; SFA: Saturated fatty acids; MUFAs: Monounsaturated fatty acids; PUFAs: Polyunsaturated fatty acids; NCEP: National Cholesterol Education Program; PGE1: Prostaglandins E1; PGI2: Prostacyclins I_2_.

## Competing interests

The authors declare that they have no competing interests.

## Authors’ contributions

AH drafted the manuscript and was involved in study design and performed statistical and data analysis. HM carried out sample collection and storage and participated in sample preparation and analysis. LU participated in results discussion and manuscript preparation. VM participated in results discussion and manuscript preparation. AM contributed to the platelet results discussion. AC participated in the coordination of patients recruitment. GT participated in the manuscript preparation. NM participated in the planning and organization of the study, in the experimental work and manuscript preparation. All the authors have read and approved the final manuscript.
